# Biomechanical Effect of Coronal Alignment and Ligament Laxity in Total Knee Arthroplasty: A Simulation Study

**DOI:** 10.3389/fbioe.2022.851495

**Published:** 2022-04-11

**Authors:** Jaehun Ro, Du Hyun Ro, Yeokyung Kang, Hyuk-Soo Han, Choongsoo S. Shin

**Affiliations:** ^1^ Central R&D Center, Corentec Co., Ltd., Seoul, Korea; ^2^ Department of Orthopaedic Surgery, Seoul National University College of Medicine, Seoul, Korea; ^3^ CONNECTEVE Co., Ltd, Seoul, Korea; ^4^ Department of Biomedical Engineering, Yonsei University, Seoul, Korea; ^5^ Department of Mechanical Engineering, Sogang University, Seoul, Korea

**Keywords:** knee arthroplasty, model, coronal alignment, collateral ligament tension, contact force

## Abstract

The purposes of this study were to develop a cruciate-retaining total knee arthroplasty musculoskeletal model, which enables the adjustment of ligament length and implant alignment; validate the model; and evaluate the effects of varus/valgus alignment adjustment and unbalanced medial/lateral ligament laxity during gait. A cruciate-retaining total knee arthroplasty musculoskeletal model was constructed and validated against the *in vivo* contact forces. This model was transformed to 2° varus/valgus alignment of femoral or tibial replacement models and 2° medial/lateral laxity models. The contact forces and ligament tensions of the adjusted models were calculated. The contact forces in the model showed good agreement with the *in vivo* contact forces. Valgus replacement alignment with balanced ligament models showed a lower contact force at the medial compartment than at the neutral alignment model, whereas the varus replacement alignment with balanced ligament models showed a greater contact force at the medial compartment and medial/posterior cruciate ligament tension. The medial laxity with neutral alignment model showed a similar contact force with decreased medial ligament tension compared to the balanced neutral alignment model, whereas the lateral laxity with the neutral alignment model showed a greater contact force and decreased lateral ligament tension. The cruciate-retaining total knee arthroplasty model was validated using *in vivo* contact forces (*r* = 0.939) Two degrees of valgus alignment adjustment with balanced ligament or neutral alignment with 2° of medial laxity can be safe without increasing contact force or ligament tension compared to neutral alignment with a balanced extension gap. However, 2° of varus alignment adjustment with balanced ligament or neutral alignment with 2° of lateral laxity may be unfavorable due to the overloading of the joints and knee ligaments.

## Introduction

The main goal of total knee arthroplasty (TKA) is providing pain relief and restoring a neutral mechanical axis (Scott, 2007). The restoration of coronal alignment and optimal gap balancing have been long-held tenets for successful TKA (Longstaff et al., 2009; Lombardi et al., 2011), and accomplishing those features has led to good long-term survival rates in TKA patients (Fang et al., 2009; Ritter et al., 2011). However, it is difficult to obtain a complete rectangular gap or neutral alignment in TKA (Fang et al., 2009; Ritter et al., 2011; Kamenaga et al., 2018). Also, the good outcomes were obtained from a neutral alignment group (mechanical axis < ± 3°) compared with a coronal alignment outliers group (mechanical axis ≥ 3°) (Fang et al., 2009; Longstaff et al., 2009; Lombardi et al., 2011; Ritter et al., 2011). However, with this classification, it is unable to compare the biomechanical effects between 2° of varus/valgus alignment and neutral alignment groups. Therefore, the biomechanical effect of 2° varus/valgus alignments remains unclear.

Currently, most surgeons permit an unbalanced gap (≤ 2°) or perform additional varus/valgus (≤ 2°) bone cuts to obtain a balanced gap. Constitutional varus was proposed in the 2010s and showed that the under-correction of the varus alignment resulted in excellent clinical outcome scores (Vanlommel et al., 2013). Moreover, the kinematic alignment has been introduced to restore the joint line of pre-arthritic knees, even though the knee alignment can be out of the neutral alignment range (Howell et al., 2013). Also, robotic-assisted surgery increased the accuracy of targeting neutral mechanical alignment by reducing alignment errors within 1°–3° compared to surgery using conventional instruments (Bellemans et al., 2007; Song et al., 2013), enabling more precise targeting of specific alignment by subtle degrees.

Previous studies have focused on evaluating the effect of alignment outliers (Fang et al., 2009; Longstaff et al., 2009; Lombardi et al., 2011; Ritter et al., 2011; Lerner et al., 2015; Kang et al., 2017; Lee et al., 2018; Nishitani et al., 2019). Various studies using computational methods evaluated the contact stress and force at more than 3° of varus/valgus alignment. Lerner et al. (2015) constructed a dynamic simulation model and calculated knee contact forces up to 8° varus- and valgus-aligned TKA. A musculoskeletal knee simulator model was used to evaluate the knee contact force and kinematics of a 7° valgus alignment model while providing 4 mm of slack on the medial collateral ligament (MCL) during deep-knee bend cycles (Nishitani et al., 2019). Therefore, evaluating the biomechanical effects of moderate varus and valgus alignments of less than 3° is necessary.

Ligament balancing is critical for maintaining knee stability and achieving equal medial/lateral gaps, as well as extension/flexion gaps in TKA (Mitsuyasu et al., 2011). Blakeney et al. (2020) reported that coronal alignment correction caused ligament imbalance. From this clinical point of view, it is necessary to construct a model containing ligaments in the knee joint for a better understanding of the biomechanical effects of balance/imbalance or medial/lateral laxity in the coronal alignment in TKA. However, previous studies did not fully reflect ligament balancing in the simulation models. Many studies have made efforts to develop TKA musculoskeletal models to predict the *in vivo* contact force (Lerner et al., 2015; Marra et al., 2015; Jung et al., 2016; Smith et al., 2016). However, some of them did not include ligament models (Lerner et al., 2015; Jung et al., 2016). Only a limited number of studies introduced their models including ligament elements and evaluated the biomechanical effect of coronal alignment outliers (Thelen et al., 2014; Chen et al., 2015; Smith et al., 2016). Hence, it is worthwhile to investigate the effect of coronal alignment changes using a simulation model that includes a detailed ligament balancing process.

Therefore, the purposes of this study were 1) to develop a cruciate-retaining (CR) TKA musculoskeletal model, which enabled the adjustment of ligament length and implant alignment; 2) validate the model; and 3) evaluate the effects of varus/valgus alignment adjustment and unbalanced medial/lateral ligament laxity on contact force and ligament tension during gait.

## Methods

### Experimental Data

The data used in this study were from the Fourth Grand Challenge Competition to Predict *In Vivo* Knee Loads (Fregly et al., 2012). The experimental data of one male subject (age 83 years, height 168 cm, body weight 66.7 kg) who received a TKA in his right knee with a telemetric knee prosthesis (D’Lima et al., 2005) were obtained when performing an overground gait trial and a static trial from the aforementioned dataset. The articulating surface of the telemetric implant geometry was based on the CR-type SIGMA^®^ (DePuy Synthes, Warsaw, IN, United States) device. The anterior cruciate ligament was removed during the TKA. *In vivo* medial and lateral contact forces measured in the prosthesis were used to validate the contact force calculated in the simulation model. The accuracy of the telemetric implant system was previously validated (D’Lima et al., 2005). The marker-based motion capture data of 41 markers in the static trial in the Grand Challenge dataset were used, and 33 markers in the overground gait trial were used. The static trial was used to predefine the marker location in the local coordinate system. The gait trial marker data were used to define the kinematics of the motion including segment location and joint angle. The motion capture data were obtained at 120 Hz and low-pass-filtered at 5 Hz. The ground reaction force data were obtained from three force plate systems. The force plate data were captured at 1,200 Hz and low-pass-filtered at 10 Hz.

### Musculoskeletal Model

**FIGURE 1:**
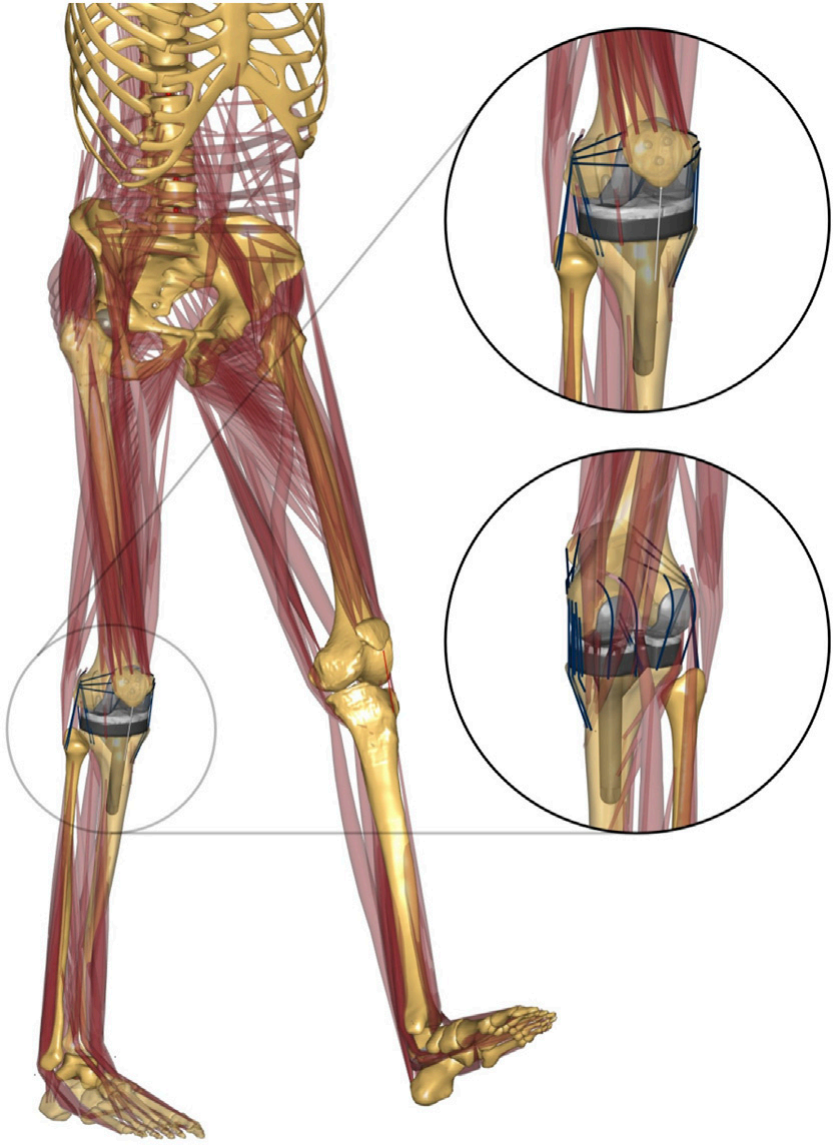
Musculoskeletal model developed in this study. Anterolateral and posterior–medial view of the TKA knee joint.

A three-dimensional musculoskeletal model was developed using the AnyBody Modeling System (V7.2.0, AnyBody Technology, Aalborg, Denmark). The AnyBody Managed Model Repository (AMMR, AnyBody Technology, Aalborg, Denmark) version 2.0 and the Twente Lower Extremity Model (TLEM, V2.0) (Carbone et al., 2015) were used as the basic models and partially modified using subject-specific bone geometries (femur, tibia, fibula, patella, pelvis, and talus) in the dataset. Subject-specific scaling of the femur, tibia, and patella were performed using affine transformation and radial basis function (RBF) interpolation transformations between the subject-specific bone and target (TLEM V2.0) bones (Marra et al., 2015), which are functions available in the AnyBody Modeling System. From this scaling process, the muscle attachment sites of the musculoskeletal model were determined. The 3D implant geometries of the tibial tray, tibial insert, femoral component, and patellar component were placed according to postoperative computed tomography images provided in the dataset. The original alignment of the bone model was neutral (mechanical axis < 0.2°). The right knee joint coordinate system consisted of a 3-degree-of-freedom (DOF) tibiofemoral joint (flexion–extension, internal–external rotation, varus–valgus rotation) and a 1-DOF patellofemoral joint (flexion–extension) in the kinematical calculation step. In the force-dependent kinematics model (Andersen and Rasmussen, 2011), the right knee joint was considered to have 6-DOF tibiofemoral joint and 6-DOF patellofemoral joint. To constrain the knee joint as a native knee joint, 21 ligament bundles were constructed as non-linear spring elements (Blankevoort et al., 1991). The modeled ligaments consisted of two bundles of anterolateral ligaments (ALLs), two bundles of lateral collateral ligaments (LCLs), five bundles of MCLs, two bundles of posterior cruciate ligaments (PCLs), four bundles of posterior capsules, three bundles of medial patellofemoral ligaments, and three bundles of lateral patellofemoral ligaments (Figure 1).

The ligament was modeled as a non-linear elastic spring using the following force–strain equations:

$$f\left(\varepsilon \right)=\{\begin{array}{cc} \frac{k\varepsilon^{2}}{4\varepsilon_{1}}, & 0\leq \varepsilon \leq 2\varepsilon_{1}\\ k\left(\varepsilon -\varepsilon_{1}\right), & \varepsilon >2\varepsilon_{1}\\ 0, & \varepsilon <0 \end{array},$$ (1)

$$l_{0}=\frac{l_{r}}{\left(\varepsilon_{r}+1\right)},\;\mathrm{and}$$ (2)

$$\varepsilon =\frac{l-l_{0}}{l_{0}},$$ (3)

where *f(ε)* is the ligament force, *ε* is the ligament strain, *k* is the ligament stiffness, and *ε*_1_ is a constant for determination of non-linear and linear phase. The ligament slack length, *l*_0_, is defined using ligament reference length *l*_*r*_, which is the ligament length at the reference position (extension). The reference strain and stiffness of each ligament bundle adapted from previous studies (Blankevoort et al., 1991; Marra et al., 2015) are summarized in Table 1.

**TABLE 1:** Reference strain and stiffness of the ligament bundles used in the musculoskeletal model (Blankevoort et al., 1991; Marra et al., 2015).

Ligament bundle	References strain^a^	Stiffness (N)^b^
aALL	0.01	2,000
pALL	0.01	2,000
aLCL	0.03	2,500
pLCL	0.03	2,500
aMCL	0.04	2,750
cMCL	0.04	2,750
pMCL	0.05	2,750
aDM	0.01	2,000
pDM	0.04	4,000
aPCL	−0.10	9,000
pPCL	−0.05	9,000
PC	0.07	1,000
sMPFL	0.07	1,200
cMPFL	0.07	1,100
iMPFL	0.07	1,000
sLPFL	0.06	1,200
cLPFL	0.06	1,100
iLPFL	0.06	1,000

aALL/pALL, anterior and posterior anterolateral ligament; aLCL/pLCL, anterior and posterior lateral collateral ligament; aMCL/cMCL/pMCL, anterior, center, and posterior medial collateral ligament; aDM/pDM, anterior and posterior deep medial collateral ligament; aPCL/pPCL, anterior and posterior cruciate ligament; PC, posterior capsule (four bundles); sMPFL/cMPFL/iMPFL, superior, center, and inferior medial patellofemoral ligament; sLPFL/cLPFL/iLPFL, superior, center, and inferior lateral patellofemoral ligament

a Reference strain was obtained at extended knee position (Blankevoort et al., 1991).

b Unit of stiffness: Newton per unit strain.

A full gait cycle was simulated based on the marker data and the ground reaction force. In the simulation model, the first step was to calculate the kinematics of the musculoskeletal model. In this step, position/rotation data of the segments and angle data of the joints were calculated. Next, the knee joint was oriented to the reference position (extended knee without any varus/valgus angle or internal/external rotation) to obtain the reference length of each ligament element. Finally, an inverse dynamics model based on the force-dependent kinematics model was driven with kinematics data calculated in the first step including the ligament reference length data from the ligament calibration process. Location data of the markers attached on the tibia and femur were used as motion input to drive the lower extremity in the inverse dynamics step. The contact of implant articulating surfaces was also considered in this step.

### Contact Force Validation

The calculated medial contact force (MCF) and lateral contact force (LCF) of the tibial insert were compared to the *in vivo* measured contact forces in the telemetric implant. The total contact force (TCF), which is a summation of the MCF and LCF, was also compared to the measured data. The accuracy of the musculoskeletal model was evaluated by quantifying the difference between the contact forces calculated from the model and the *in vivo* measured contact forces in terms of root-mean-squared error (RMSE) and Pearson’s correlation coefficient (*r*) (D’Lima et al., 2005).

### Coronal Alignment and Ligament Laxity

**FIGURE 2:**
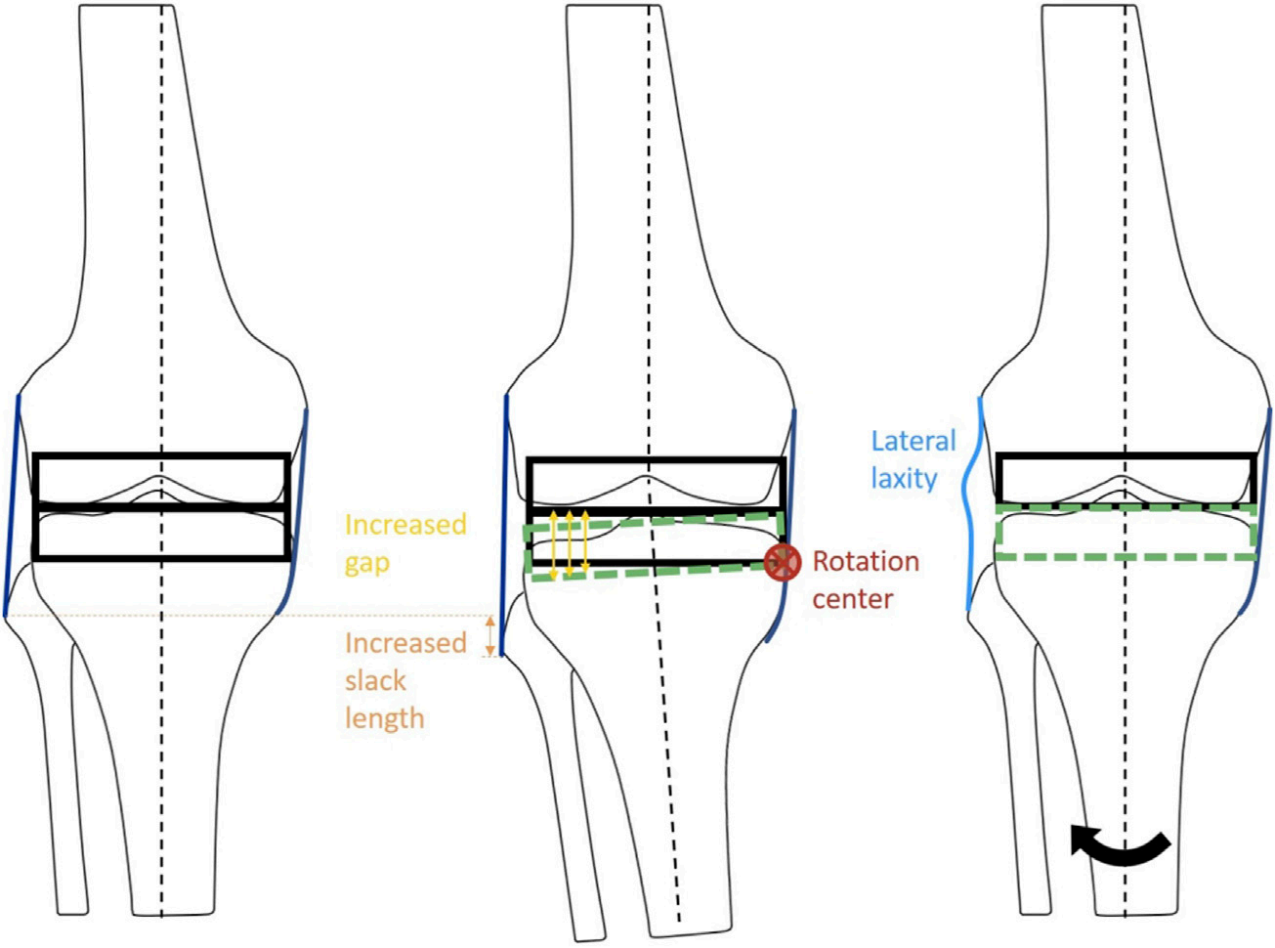
Schematic diagram of the ligament adjustment process. The balanced neutral alignment model (left). Ligament adjustment performed at 2° varus (center). Model G: Neutral alignment model with laxity at lateral ligaments with balanced medial ligaments (right). Black arrow indicates the rotation of tibia from varus to neutral alignment.

**FIGURE 3:**
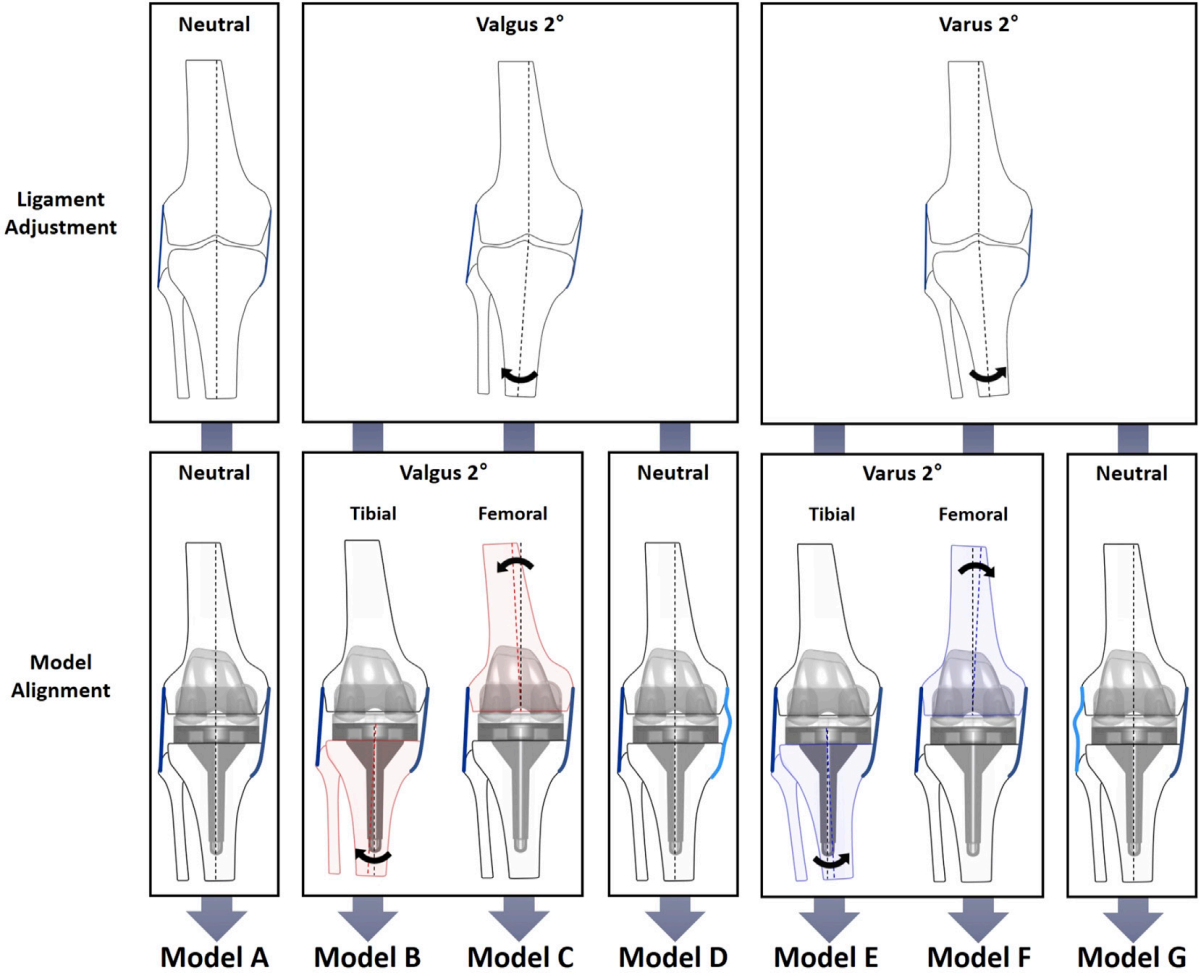
Diagram of varus/valgus, medial/lateral laxity, and tibial/femoral replacement model configurations. The neutral alignment model (model A) was transformed into six cases: the 2° tibial valgus (model B) and femoral valgus (model C) alignment models with no laxity; the 2° tibial varus (model E) and femoral varus (model F) alignment models with no laxity; and neutral alignment models with 2° medial (model D) and lateral (model G) laxity. Medial and lateral laxity in models D and G are illustrated by light blue lines.

To evaluate the biomechanical effects of the combinations of varus/valgus alignment and ligament balancing, neutral/varus/valgus alignment of the femoral/tibial components and medial/lateral ligament laxity configuration models were constructed (Figures 2, 3). Model A: neutral alignment with balanced ligaments, model B: tibial valgus alignment with balanced ligaments, model C: femoral valgus alignment with balanced ligaments, model D: neutral alignment with medial laxity, model E: tibial varus alignment with balanced ligaments, model F: femoral varus alignment with balanced ligaments, and model G: neutral alignment with lateral laxity. These models used the same input data (i.e., ground reaction force, marker data, and bone geometries) as the validation model (neutral). Only the alignment of the femoral or tibial components and ligament properties were adjusted.

The medial and lateral laxity was provided by adjusting the alignment at the ligament calibration step to simulate residual laxity by leaving 2° of gap difference (Figure 2). First of all, the ligaments surrounding the knee joint were adjusted at the neutral mechanical axis in model A, which was a balanced neutral alignment model used as a reference. The ligaments of models B, C, and D were adjusted to be balanced at a knee extension of 2° valgus alignment. In the same manner, the ligaments of models E, F, and G were adjusted to be balanced at a knee extension of 2° varus alignment. Next, models B (tibial) and C (femoral) were aligned at 2° valgus, and models E (tibial) and F (femoral) were aligned at 2° varus, while the ligaments were balanced in length in each alignment. For the neutral alignment adjustment models, medial laxity and lateral stability (model D), and lateral laxity and medial stability (model G) were provided by leaving 2° of medial or lateral gap difference, respectively.

## Results

### Musculoskeletal Model Validation

**FIGURE 4:**
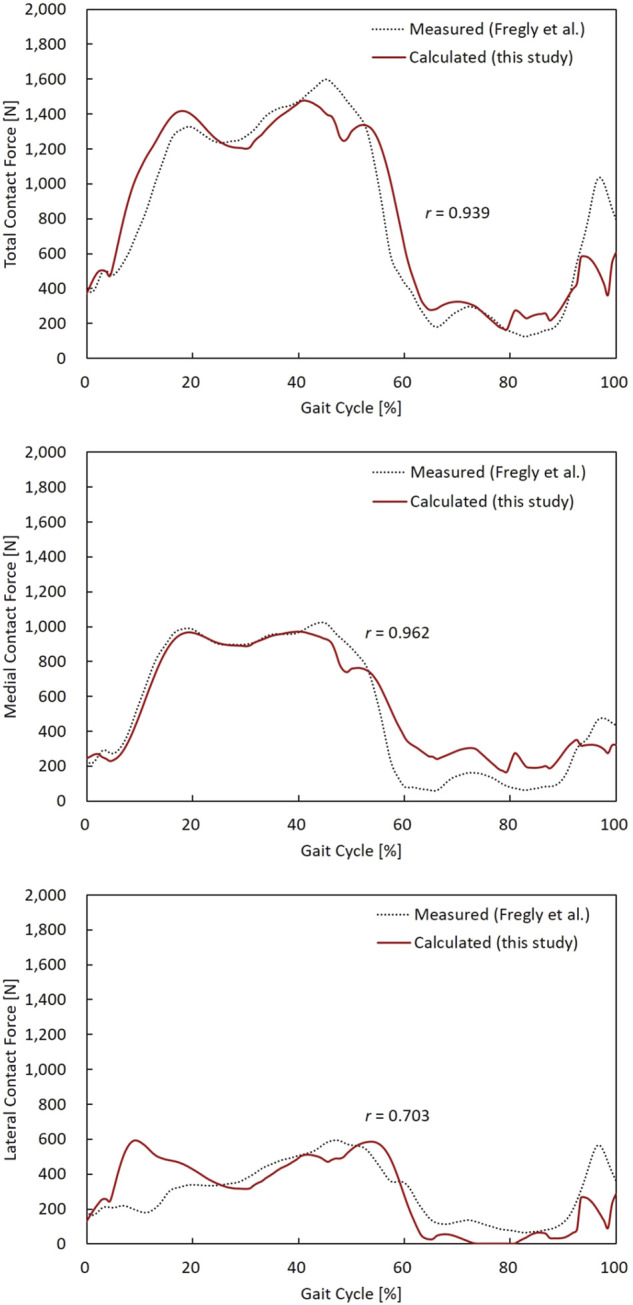
TCF, MCF, and LCF calculated from the model, and the measured data were compared during a gait cycle. Pearson’s correlation coefficient (r) for TCF, MCF, and LCF were 0.939, 0.962, and 0.703, respectively.

In general, the magnitude and pattern of the contact forces in the model showed good agreement with the measured *in vivo* contact forces (Figure 4). The RMSEs of the TCF, MCF, and LCF magnitude (% body weight (BW)) were 175 N (27.6%), 118 N (18.6%), and 147 N (23.2%), respectively. Both the TCF (*r* = 0.939) and MCF (*r* = 0.962) showed very high correlation coefficients, but lower accuracies of the LCF (*r* = 0.703) were observed in the early stance phase of the gait cycle.

### Effect of Alignment and Laxity

**FIGURE 5:**
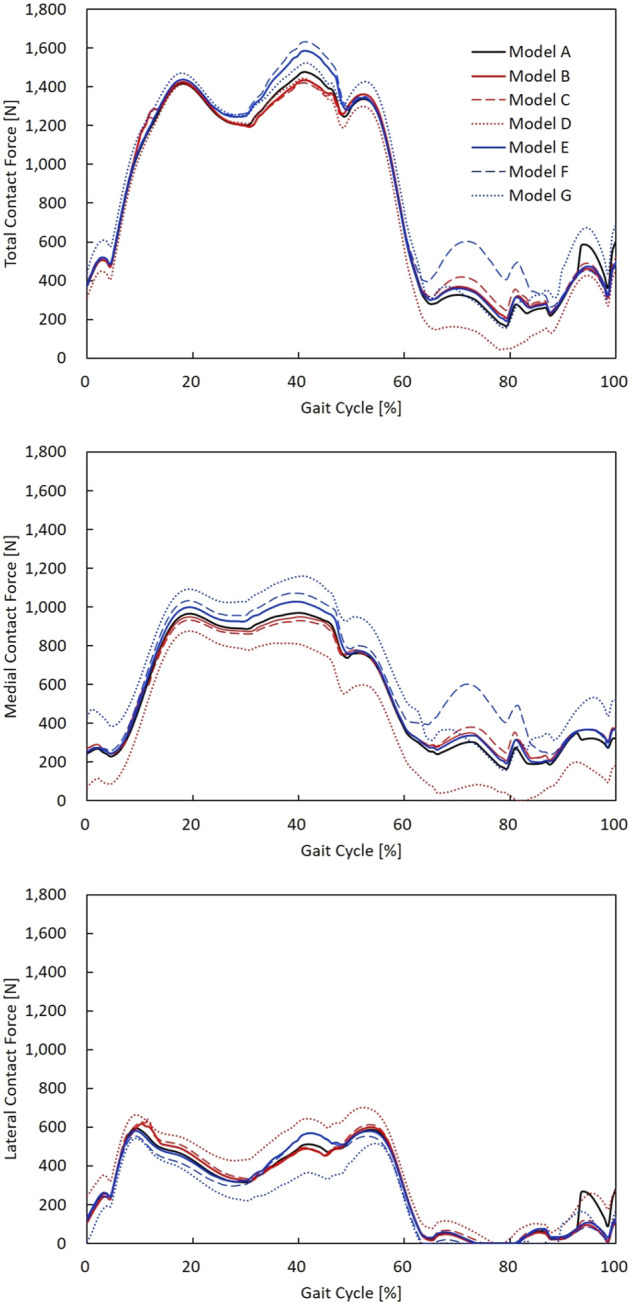
TCF, MCF, and LCF calculated during a gait cycle in models A–G. The neutral alignment model (model A); the 2° tibial valgus (model B) and femoral valgus (model C) alignment models with no laxity; the 2° tibial varus (model E) and femoral varus (model F) alignment models with no laxity; and neutral alignment models with 2° medial (model D) and lateral (model G) laxity.

**FIGURE 6:**
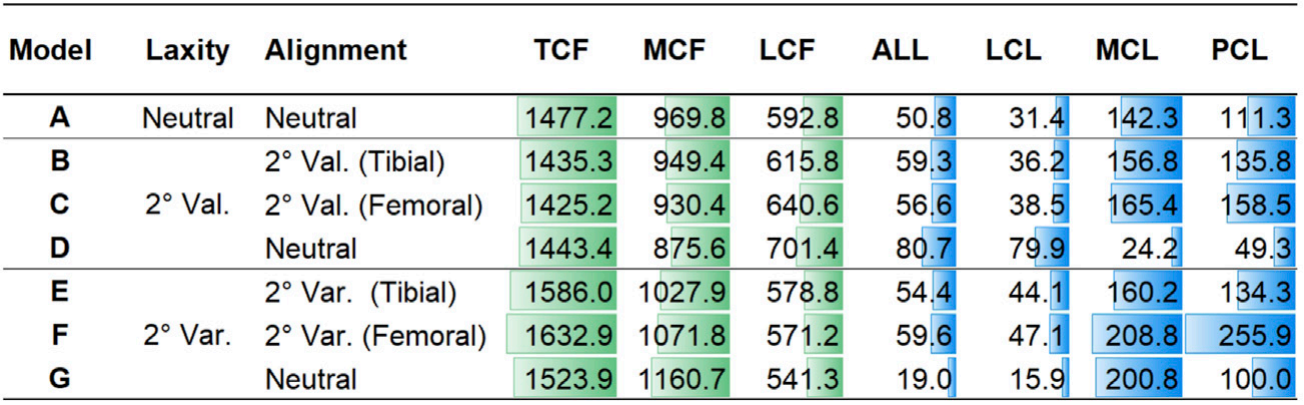
Peak TCF, MCF, and LCF and peak tension force of the ALL, LCL, MCL, and PCL in models A–G in Newton.

The contact forces during a gait cycle in varus/valgus and medial/lateral laxity model configurations are shown in Figure 5. The peak TCF, MCF, LCF, and ligament tensions are illustrated in Figure 6. Tibial and femoral valgus alignments with balanced ligament models (models B and C) showed lower TCF (−3.2%) and higher medial (+13.2%) and lateral ligament tension (+16.5%), whereas the tibial and femoral varus alignment with balanced ligament models (models E and F) showed greater TCF (+9.0%) and higher medial (+30.0%) and posterior ligament tension (+75.3%) than the neutral alignment model (model A).

The medial laxity with the neutral alignment model (model D) resulted in lower TCF (−2.3%) with lower medial ligament tension (−83.0%), and the lateral laxity with neutral alignment model (model G) resulted in greater TCF (+3.2%) and lower lateral ligament tension (−56.0%) than the neutral alignment model (model A).

The medial laxity with the neutral alignment model (model D) resulted in slightly higher TCF (+0.9%) and lower medial (−85.0%) and posterior ligament tension (-66.3%) than the tibial/femoral valgus alignment with balanced ligament models (models B and C). The lateral laxity with the neutral alignment model (model G) showed lower TCF (−5.3%) and lateral ligament tension (−65.8%) than the tibial/femoral varus alignment with balanced ligament models (models E and F).

**FIGURE 7:**
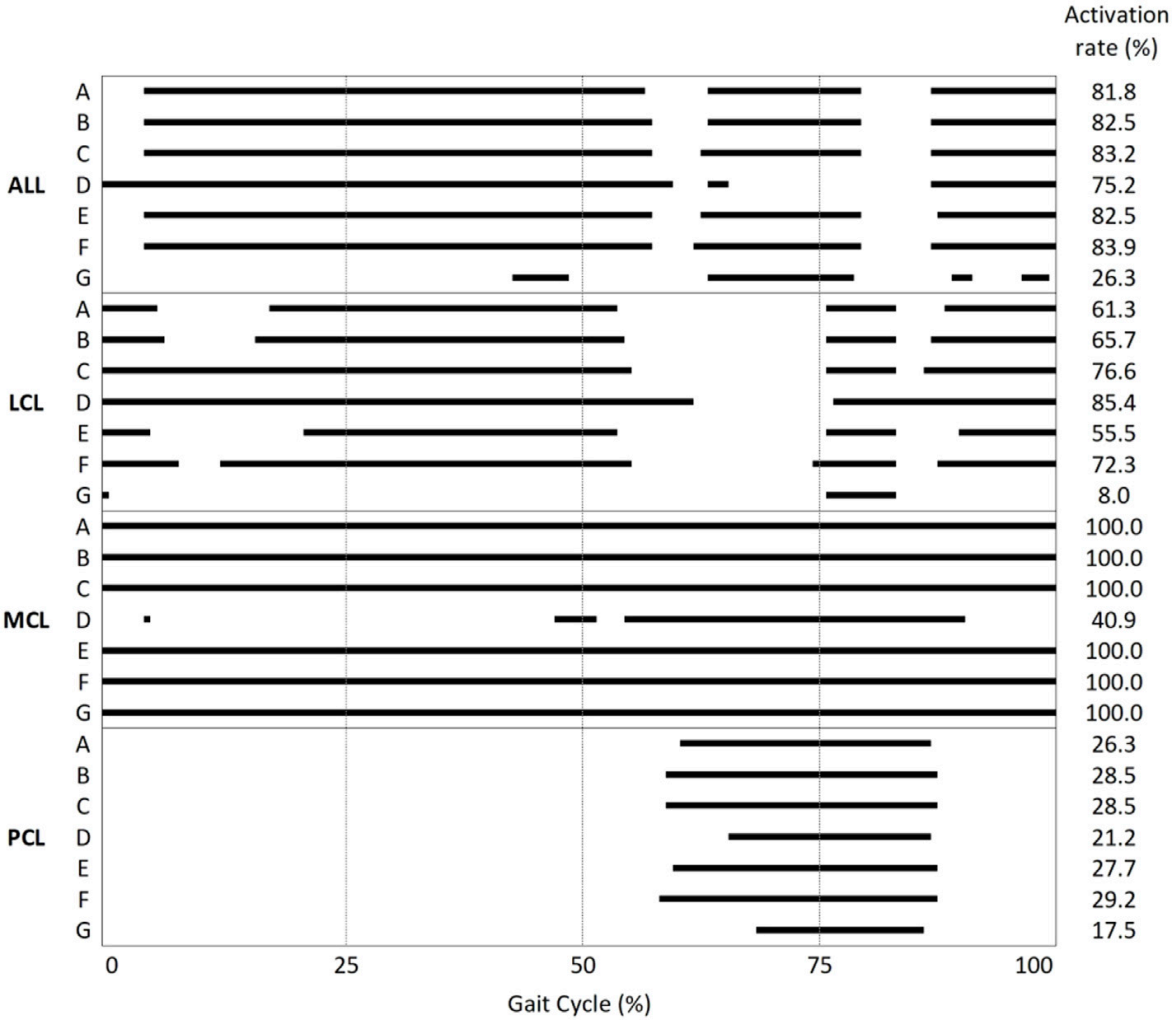
Activation/deactivation point and activation rate during the gait cycle of the ALL, LCL, MCL, and PCL in models A–G.

The ligament activation (i.e., tightening) time results represented by the percentage of gait cycle are summarized in Figure 7. The lateral laxity model with the neutral alignment model (model G) showed the lowest activation rate among all models. In every model, except model D, MCL bundles were fully activated during the gait cycle.

### Effect of Tibial and Femoral Alignment Model

The tibial varus alignment model (model E) showed lower TCF (−2.9%) than the femoral varus alignment model (model F). Decreased medial (−23.3%) and posterior ligament tension (−47.5%) were also observed. The tibial valgus alignment model (model B) resulted in a slightly higher TCF (+0.7%) than the femoral valgus alignment model (model C).

### Kinematics

**FIGURE 8:**
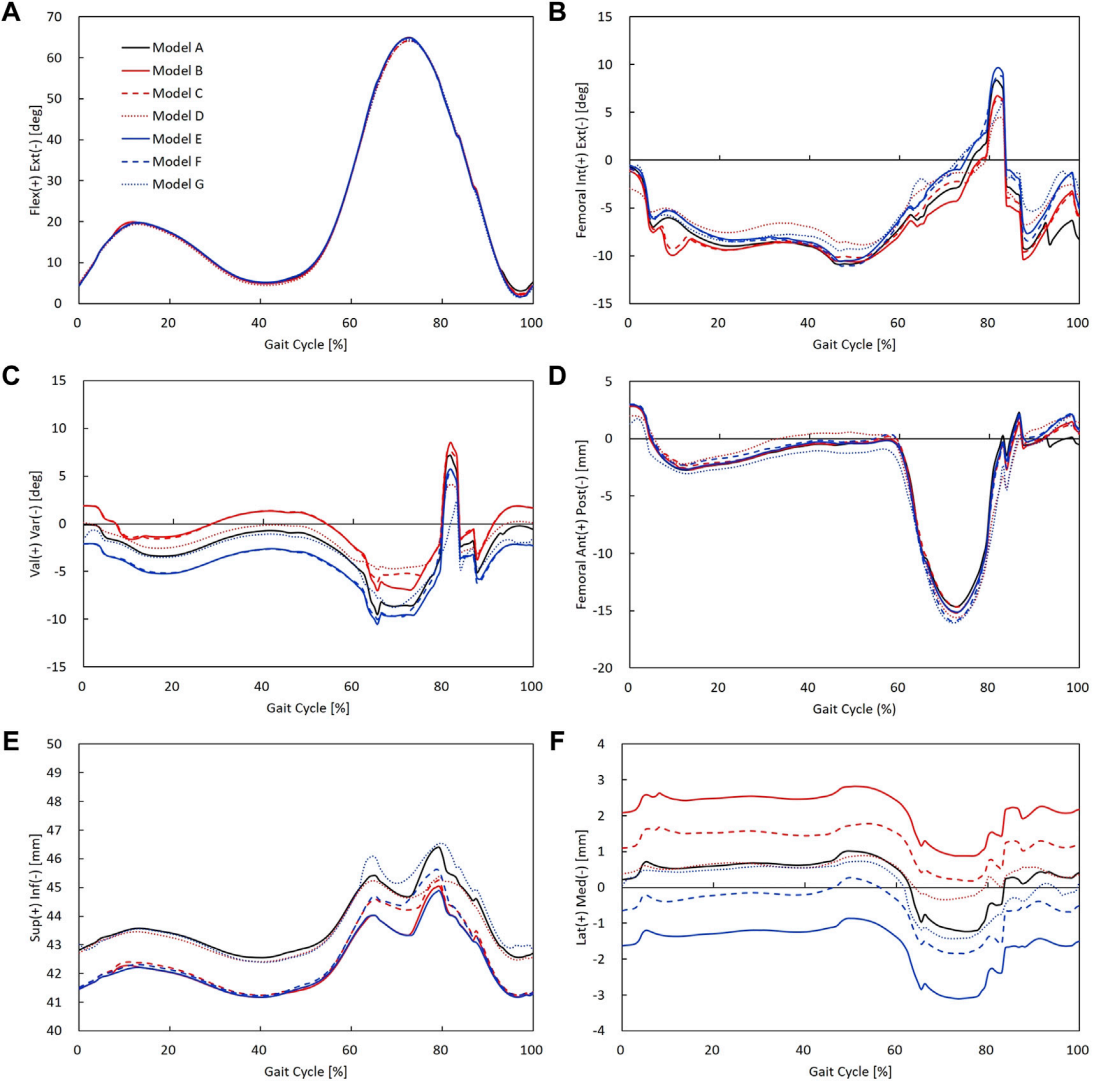
Rotational and translational knee kinematics in models A–G: **(A)** knee flexion angle, **(B)** femoral internal/external rotation, **(C)** knee varus/valgus angle, **(D)** femoral anterior/posterior translation, **(E)** superior/inferior translation (the initial value of superior–inferior translation indicates superior–inferior height difference between the tibial and femoral coordinate systems), and **(F)** femoral medial/lateral translation.

Rotational and translational kinematics of the knee joint was compared between alignment/laxity models during a gait cycle (Figure 8). Six DOFs were all calculated for the femoral coordinate system with respect to the tibial coordinate system. The maximum flexion angle difference between alignment/laxity models and the neutral alignment model was less than 1° (Figure 8A). Maximum posterior translation in the varus/valgus alignment and medial/lateral laxity models increased compared to the neutral alignment model (Figure 8D). Varus alignment models translated more laterally, while the valgus alignment model translated more medially (Figure 8F). The tibial varus/valgus alignment showed to shift more than the femoral varus/valgus alignment. However, neutral alignment models with medial/lateral laxity showed a less difference (< 0.5 mm) than the varus/valgus alignment in the stance phase compared to the neutral alignment model.

## Discussion

The accuracy of the estimated contact forces compared to the *in vivo* data was equivalent to or higher than that in previous studies. The RMSEs of the TCF, MCF, and LCF were less than 0.28 BW during one gait trial. To the best of our knowledge, the TCF RMSE was lower in all previous studies, except for one (range, 0.26 BW—0.51 BW) (Marra et al., 2015). In particular, the RMSE of the MCF (0.19 BW) in our study demonstrated that the developed model well-predicted the quantitative contact force compared to previous studies (range, 0.21 BW—0.26 BW). Likewise, the RMSE of the LCF in the present study was 0.23 BW, while the RMSEs of the MCF in recent studies ranged from 0.22 BW to 0.42 BW (Jung et al., 2016; Marra et al., 2015; Smith et al., 2016; Thelen et al., 2014) (Table 2). In addition, our results showed that the magnitude of the contact force in the medial compartment accounted for 65.5% of the TCF, which agrees with the previous findings that 64.2 ± 6.0% of the TCF passes through the medial compartment during walking (Werner et al., 2005). We were able to develop an accurate model by benchmarking the features modeled in the two most accurate previous studies, which minimized errors in the marker location between the model and the experimental trial by using a reference trial (Marra et al., 2015) and releasing the constraint of the knee joint to a spherical joint during the kinematics calculation (Jung et al., 2016). Therefore, the computational TKA model developed and analyzed in this study seemed to sufficiently predict contact forces and assess the effect of ligament tension by varying the coronal alignment of the TKA during a gait cycle.

**TABLE 2:** Model accuracy of this study compared to previous studies.

	TCF	MCF	LCF
	RMSE (BW)	RMSE (BW)	RMSE (BW)
Our study	0.28	0.19	0.23
Jung et al. (2016)	0.38	0.21	0.26
Marra et al. (2015)	0.26	0.26	0.35
Thelen et al. (2014)	0.51	0.26	0.42
Smith et al. (2016)	0.33	0.23	0.22

TCF, total contact force; MCF, medial contact force; LCF, lateral contact force; RMSE, root-mean-squared error; BW, body weight.

The main observation of this study was that 2° valgus alignment adjustment with balanced ligament (models B and C) or neutral alignment with 2° medial laxity (model D) decreased the contact force compared to the balanced neutral alignment. In contrast, 2° of varus alignment adjustment with balanced ligament (models E and F) or neutral alignment with 2° lateral laxity (model G) increased the contact force and ligament tension compared to the balanced neutral alignment. The TCF is simply a summation of the MCF and LCF. Since the MCF constitutes a larger proportion of the TCF than LCF (Werner et al., 2005), the reduction of MCF in models B, C, and D may have caused the reduction in TCF. In the same manner, the increase in MCF in models E, F, and G may have caused the increase in TCF. Similar tendencies of MCF and LCF values were observed in a previous study that evaluated the effects of varus/valgus outliers more than 2° (Chen et al., 2015). Also, the results of the finite element analysis study showed that the medial contact stress change was larger than the lateral contact stress change in the varus/valgus alignment (Suh et al., 2017). Increased contact forces in TKA may lead to severe wear and result in early revision (D’Lima et al., 2001; Srivastava et al., 2012). Therefore, 2° of valgus alignment adjustment with balanced ligament or neutral alignment with 2° medial laxity may be safer than neutral alignment, whereas varus alignment adjustment with balanced ligament or neutral alignment with 2° lateral laxity may not be beneficial in terms of implant survivorship.

It is interesting to note that neutral alignment with ligament adjustment in 2° valgus (model D) or varus (model G) alignment showed large reductions in MCL or LCL tension, respectively, while stabilized ligament tension was observed in the opposing side. Suh et al. (2017) reported that 3°–5° varus alignment led to excessive decreases in MCL force but no remarkable change in the ALL and LCL force. However, they did not describe whether the ligaments were balanced or not. The discrepancy between the results of our study and the previous study may be inferred from the presence or absence of novel laxity adjustment in the models. Not only the peak tensions of the MCL and LCL decreased in medial and lateral laxity but also the ligament activation (i.e., tightening) time decreased compared to that of neutral alignment. The MCL in the medial laxity model (model D) was activated in only 40.9% of the gait cycle, whereas in other neutral alignment models, the MCL was activated during the entire gait cycle. Likewise, the LCL in the lateral laxity model (model G) was activated in only 8.0% of the gait cycle, whereas in other neutral alignment models, the LCL was activated in 55.5–85.4% of the gait cycle. Since the medial (model D) and lateral (model G) laxity models give slack to the medial and lateral soft tissues, respectively, ligament tension on the lax side will be loaded later and unloaded earlier. In this regard, we verified that MCL and LCL slack were provided properly in the medial (model D) and lateral (model G) laxity models.

The results of this study showed that the contact force of the femoral varus alignment was 2.9% greater than that of the tibial varus alignment. The greater contact force in the femoral than the tibial varus alignment observed in our study may have resulted from increased MCL and PCL tension (Figure 6). Our findings agree with a previous simulation study that reported that the maximum contact force in a 5° femoral varus alignment was 6.2% greater than that in the tibial varus alignment (Chen et al., 2015). Lee et al. (2018) conducted a long-film study evaluating the failure mechanism of femoral/tibial malalignment and found that femoral varus alignment had an increased mechanical failure rate, consistent with our results. In contrast to the results that tibial varus/valgus alignment can be safer than femoral varus/valgus alignment, Innocenti et al. (2016) showed that 2°–6° femoral varus/valgus alignment resulted in lower stress than those of tibial malalignment, although the model they used included only MCL and LCL without other ligaments or soft tissues in the knee joint. A short-film study reported inferior outcomes in the tibial varus alignment compared to the femoral varus alignment (Ritter et al., 2011). Due to wide variations in the femoral shaft bowing in the coronal plane (Kim et al., 2015), the anatomical axis evaluated with short films may be insufficient compared to the evaluation of the mechanical axis in long-film studies. Altogether, our results comparing femoral and tibial varus alignments indicate that the femoral varus alignment should be avoided in case 2° of the varus alignment adjustment is necessary for a balanced ligament.

The kinematics results in this study showed that medial/lateral laxity did not affect joint instability. Our results showed that internal/external rotation decreased due to medial or lateral laxity during gait. These results agree with a previous cadaveric study that reported MCL release reduced the internal rotation of the tibia during flexion (Wada et al., 2017). Takagi et al. (2021) also reported that intraoperative medial and lateral laxity influenced the rotational kinematics during a deep knee bend activity. In contrast, valgus alignment showed to rotate more internally and varus alignment showed to rotate more externally than the neutral alignment model (Figure 8B). The alignment differences of the varus/valgus alignment were well reflected in the results of varus/valgus angle (Figure 8C). These findings agree with a previous study reported that valgus alignment of the tibiofemoral joint was shifted toward its alignment direction (Thelen et al., 2014). In addition, varus/valgus alignment models showed to translate inferiorly compared to the neutral alignment model. As varus and valgus models were medially and laterally overresected, respectively, this may have resulted in decreased knee joint space compared to the neutral alignment model. Consequently, there existed changes in kinematics influenced by coronal alignment change, whereas comparable kinematics of 2° medial/lateral laxity models were observed compared to the balanced neutral alignment model.

This study had some limitations that should be considered when interpreting the results. First, the model was validated by one participant performing a gait trial. Gait motion is the most frequently performed activity in daily living. Nevertheless, the knee flexion angle varies from 0° to 65°, and it is not appropriate to predict tibiofemoral contact force at high flexion beyond 65°. Second, the subject in this study received a TKA with a CR-type tibial insert. Different insert options, such as a posterior-stabilized (PS) type, may result in different biomechanical behaviors. A previous study reported that joint gaps differed between PS and CR-TKA (Matsumoto et al., 2009). Third, only the contact forces were analyzed at the articulating surface. For better understanding the phenomenon in the view of structural mechanics, it may be necessary to check pressure or stress distribution through structural analysis. Fourth, the varus/valgus alignment more than 2° was not considered in this study. Fifth, previous studies (Suh et al., 2017; Marra et al., 2018a; Marra et al., 2018b) and the current study performed validation of the musculoskeletal model prior to evaluating the alignment in TKA. However, the modified musculoskeletal models used to evaluate the effects on alignment were based on the motion of the subjects’ alignment but not on the motion of the varus/valgus alignment limb. Our model was based on a subject within average age, height, and body weight for TKA. Nevertheless, one has to be cautious when generalizing the results from this study to all TKAs. Sixth, the kinematics such as internal/external rotation of this model was not validated against *in vivo* kinematics; thus, it must be considered while interpreting the kinematic results of this study. In this study, we evaluated the effect of varus–valgus 2°, which was within the outliers, and included ligament laxity adjustment in the model to mimic more realistic situations.

In conclusion, the present study developed a musculoskeletal model to predict the contact force of CR-TKA during walking that enabled ligament laxity adjustment for coronal alignment changes, and the model was validated using *in vivo* contact forces. We found that 2° of valgus alignment adjustment with balanced ligament or neutral alignment with 2° medial laxity could be safe without increasing the contact force or ligament tension. However, 2° of varus alignment adjustment with balanced ligament or neutral alignment with 2° lateral laxity may be unfavorable due to biomechanical overloading of the joints and knee ligaments. In addition, excessive loading was observed in the medial and posterior ligaments in femoral varus alignment. Hence, surgeons should be cautious in making decisions involving tibial/femoral alignment with 2° of varus/valgus alignment.

## Data Availability

Publicly available datasets were analyzed in this study. These data can be found at: Fourth Grand Challenge Competition to Predict in vivo Knee Loads (https://simtk.org/projects/kneeloads).
